# Consumer trust and willingness to pay for establishing a market-based animal welfare assurance scheme for broiler chickens

**DOI:** 10.1016/j.psj.2023.102765

**Published:** 2023-04-29

**Authors:** Katriina Heinola, Terhi Latvala, Jarkko K. Niemi

**Affiliations:** ⁎ Natural Resources Institute (Luke), FI-00790 Helsinki, Finland; † Natural Resources Institute Finland (Luke), FI-60320 Seinäjoki, Finland

**Keywords:** willingness to pay, welfare, broiler, food labeling

## Abstract

The aim of this study was to examine Finnish consumers’ preferences and willingness to pay (**WTP**) for measures to improve broiler chicken welfare and to examine how familiarity with animal production farms, trust in food system actors, and views concerning the responsibility for animal welfare were associated with the WTP. A survey instrument, including a choice experiment, to study consumer preferences for food quality attributes was developed. The survey data were analyzed using a latent class model and logistic regression analysis.

The results suggested that Finnish consumers tend to have a positive WTP for improvements in farm animal welfare (**FAW**) in broiler production, although WTP varied by consumer segment. Five different consumer groups with different levels of WTP for specific welfare attributes were identified. The highest WTP was estimated for an additional space allowance for the birds and enhanced litter quality monitoring at the farm.

In conclusion, there is demand for animal welfare labeled broiler chicken among Finnish consumers. However, consumer expectations are heterogeneous, and different strategies can appeal to different segments. While one consumer segment prioritizes FAW over price, another segment emphasizes low-priced products and considers mid-market products appealing. Moreover, public policies are an important tool for enhancing FAW for a large proportion of consumers, and public actions are therefore warranted. Finally, engaging with animal protection organizations (as mediators) and being open to consumers can be an effective strategy to build confidence in premium products.

## INTRODUCTION

The sustainability of farm animal production has gained attention in recent years. The public would like to know more about how animal-based food is being produced, and an increasing share of Europeans considers that the choice of high animal welfare food available in grocery stores is inadequate (TNS Opinion and Social, 2015). Hence, as part of the quality product differentiation (Buller et al., 2018), voluntary farm animal welfare (**FAW**) assurance schemes which respond to the demand for higher FAW have been introduced. The schemes are related to the social and economic sustainability of farming through the morality of action of animal production and higher payments for producers.

It is important to discover, which welfare aspects consumers believe are important for FAW and could be highlighted in terms of marketing (de Jonge and Van Trijp, 2013) and in actions taken to improve FAW. Poultry is one of the production lines, which has been criticized for the inadequate FAW. For example, the housing conditions of broilers and laying hens have been criticized worse in relation to FAW than those of other farmed animals (Harper and Henson, 2001; Clark et al., 2016). Facilitating species-specific behavior for example by providing enrichments represents a dimension of animal welfare along with more health-oriented attributes, such the litter quality. Consumers’ interest in FAW seem to elaborate natural behavior and living environment rather than animal centered welfare issues such biology or behavior (Evans and Miele, 2008; Heerwagen et al., 2013). While organic production provides higher standards in terms of FAW, for example, reduced stocking density and other resources offered to the birds, the market share of organic broiler meat is in many countries marginal. For example, in Finland only about 0.2% of broiler meat production is organic (Niemi and Väre, 2019), and it is substantially more expensive, with consumer prices up to 4 times that of a conventional broiler. In fact, so-called mid-market production may be more cost-effective way of taking production animal welfare attributes (see Gocsik et al., 2016). Hence, there is room for more affordable but high animal welfare broiler products in the market.

FAW assurance schemes bring costs to the actors in the food chain, especially to the primary production and processing if actors need to invest in novel production practices to meet the requirements of the FAW assurance scheme. Hence, the supply chain actors expect higher prices from the markets to cover these additional costs. In several studies and for various animals (e.g., Umberger et al., 2002; Elbakidze and Nayga, 2012; Li et al., 2016), including broiler chicken (Makdisi and Marggraf, 2011; Mulder and Zomer, 2017; Yang and Hong, 2019), a positive willingness to pay (**WTP**) for higher FAW and support for market-based solutions has been found. In a meta-study of Clark et al. (2017), differences in WTP for FAW between animal type, region, and population of interest, and also person's knowledge of FAW practices, which is rather low, were found. “Knowledge” can be based on facts learned or experienced or on attitudes which may arise also from misconceptions concerning animal farming practices (Clark et al., 2016).

However, it has been questioned whether the WTP for FAW is sufficiently high, and whether consumers bear the (sole) responsibility for improving FAW (Lundmark et al., 2018). The concept of responsibility includes both the responsibility to ensure the desired FAW in practice and the responsibility to bear the costs of this. Consumers’ trust in actors is essential, because mistrust is one of the factors leading to a consumer not to purchase FAW-labeled products (Clark et al., 2016). The public considers veterinarians, animal health authorities, animal welfare organizations, quality assurance schemes, and consumer organizations being among the most trusted organizations and the trust on authorities and farmers tends to be highlighted in the Northern Europe (Nocella et al., 2010; Clark et al., 2019). While traditional marketing approaches in which a price premium is paid for the product are commonly accepted, there may be innovative solutions such as a mutual industry fund or crowd funding as ways for the public to assume financial responsibility of FAW improvements (Yrjölä et al., 2020). It is less well known how the financial responsibility for FAW assurance schemes should be borne, and who should bear it, and how the sense of responsibility contributes to the consumer's WTP for FAW assurance.

Against this background, the aim of this study was to examine i) Finnish consumers’ preferences for higher broiler welfare criteria by assessing WTP, including the various measures related to health and behavioral needs, and ii) the association of WTP with the trust and financial responsibility of various actors. The WTP for broiler produced to different FAW standards was examined by using a choice experiment. The heterogeneity of respondents in the sample was examined by a latent class model and logistic regression, which offered the possibility to examine diverse consumer segments and factors associated with consumers’ preferences. This study contributes to the literature with an analysis of how the consumer's trust in certain actors as information sources of FAW and the opinion concerning the responsibility related to the financing of the FAW label system were associated with WTP for various FAW measures in different consumer segments. The information can be utilized to identify the role of policy and business (e.g., marketing strategies) in developing the FAW label system.

## MATERIALS AND METHODS

### Data Collection

The choice experiment's data were gathered using a consumer survey instrument designed by the research team. Before the start of the data collection, the survey was piloted in a group of respondents (*N* = 60), with the aim of testing and refining the properties of the experimental design and enhancing the overall understanding of the survey format by the consumers. To collect the data, the survey instrument was distributed by a market research agency as an online survey to a random sample of citizens in Finland in September 2018. The survey data used in the current study included responses from *N* = 400 citizens, representing the population of Finland. The pilot study's respondents were recruited using the consumer panel of Taloustutkimus Ltd. (Helsinki, Finland), whereas the main sample was collected by Makery Ltd (Helsinki, Finland).

### Survey Design

In the survey, respondents’ opinions regarding the development of FAW and current status of broiler welfare, the importance of poultry welfare measures, respondents’ connections with and knowledge of farming, opinions on the preferred financing approach for a potential FAW labeling system, and respondents’ trust in certain actors as the source of information on FAW and FAW labeling were sought. The specific FAW attributes investigated were selected based on a scientific literature review related to broiler welfare and the stakeholders’ opinions on the most relevant requirements for welfare, which ensured the true relevance of the measures. Additionally, the FAW attributes were required to be feasible to implement by the producers, which challenged welfare indicators measured from the animals when compared to the resources-based measures such as additional space allowance (Knierim and Winckler, 2009).

Because the intention is to introduce the welfare attributes to consumers when launching a new product to the market, it is preferred to select the attributes from the perspective of a “positive story.” For example, rate of mortality or food pad damages won't appeal to consumers and represents a negative image, even though they provide information on the state of animal (Johnsen et al., 2001). The FAW attributes evaluated in the experiment were 1) reduced stocking density, 2) enrichment material such as ramps and levels, 3) higher litter quality, and 4) advanced foot health monitoring at the farm level. A slow growing breed was also examined in the pilot choice experiment. However, it was excluded from the final design, because it remained statistically insignificant in the pilot data, and the fewest respondents considered it a criterion that should be included in a FAW label for broiler meat.

Foot pad dermatitis (**FPD**) and hock skin lesions are common causes of painful lameness in broilers, which can be reduced by better litter quality (Dawkins et al., 2004; Allain et al., 2009). In addition, chicken mortality is reduced (Dawkins et al., 2004). Litter moisture is the primary factor to cause the development of foot pad lesions (Eichner et al., 2007). Providing environmental enrichments has a positive effect on welfare by increasing the behavioral opportunities and leading to improvements of biological function (Riber et al., 2018). Reduced stocking density has many positive effects on overall welfare, for example, decreased locomotion and ground pecking, incidence of leg problems, and daily mortality (Hall, 2001). The levels of the attributes used in the choice experiment are shown in Table 1. The consumer price of organic chicken can be up to 4-folded compared to the conventional broiler, which was taken into account in the price premium range, that is, the highest price premium compared to the price of organic chicken.

**Table 1:** Welfare attribute and price premium levels used in the choice experiment enquiring about consumer willingness to buy a 400 g package of broiler fillet.

Attributes	Levels
Stocking density	Level 1 Current stocking density, 18 birds per m²
	Level 2 More space, 16–17 birds per m²
	Level 3 Even more space, 15 birds per m²
Enrichments	Level 1 No enrichments required
	Level 2 Ramps and levels
	Level 3 Ramps, levels and dust bathing areas
Litter quality	Level 1 No requirements for litter
	Level 2 Litter monitored at the farm level with an action plan for improvements when anomalies are observed
Foot health	Level 1 No requirements for foot health monitoring at the farm
	Level 2 Foot health monitored at the farm and an action plan for improvements when anomalies are observed
Price premium	Product price €4.00 *plus* a premium representing one the following options:
	€1.00, €2.00, €3.00, €5.00, €7.50, €10.00, €12.00.

Before presenting the choice cards to the respondent, the survey introduced the welfare measures related to broiler production by explaining their welfare effects. The current price of a standard unflavored broiler fillet, about €4.00 per 400 g packing at the time of the survey (i.e., corresponding to €10 per kg) was presented to the respondents to remind of the average price of the product. Before entering to the choice cards, it was carefully showed with an example and explained that the price premium was in addition to the average price (“for example, with price premium of €5.00, the broiler fillet would cost €9.00 in total, considering that the average price is about €4.00”). For each respondent, 3 different product alternatives and the opt-out-alternative (“I would not choose any of the proposed products”) were presented to consider respondents not eating broiler products or preferring other product attributes than those provided.

The pilot study included 12 choice sets in 2 blocks and provided priors for the attribute levels. The Ngene software (v 1.2.1) was used to generate an efficient D-design that optimizes the use of each attribute (Rose and Bliemer, 2009) with Gaussian draws. The D-error for the final design was 0.032. The Bayesian priors were used for enrichments and litter quality, and the fixed priors for other attributes based on the statistical significance of the coefficients from the pilot study. In the main survey, there were 36 different choice sets in total and the respondents were divided into 6 blocks. Each respondent was presented with 6 choice sets and there were 3 different product specifications per choice set.

### Estimation of Willingness to Pay

The choice experiment utilizes the theory of value and the random utility theory (Lancaster, 1966). The method has been applied especially in environmental economics, transport, and consumer studies (e.g., Cicia and Colantuoni, 2010; Pouta et al., 2014; Arencibia et al., 2015). Models estimated based on choice experiment data can be used to derive respondents’ WTP for studied product attributes. Based on random utility theory, it was assumed that the respondents chose the alternative that yielded the highest utility to the person choosing between different products (McFadden, 1974). The overall utility obtained from a good can be decomposed into attributes as:

$$U_{in}\left(Z_{in}\right)=V_{in}\left(Z_{in}\right)+\varepsilon_{in}$$

The utility ($U_{in}$) is composed of 2 parts: A deterministic and observable component (*V_in_*), which is influenced by product-specific attributes (*Z_in_*); and a stochastic component (*Ɛ*_in_). It is considered that a respondent *n* chooses alternative *i* that yields the highest utility to her/him in the given choice set. *V_in_* is the systematic utility, which is the function of explanatory variables and the unobserved random parameters *β*, which represent marginal utility. The random utility model can be presented as:

$$U_{in}\left(Z_{in}\right)=V_{in}\left(Z_{in}\right)+\varepsilon_{in}=\beta^{\prime }x_{in}+\varepsilon_{in},$$

where *β* represents a vector of unknown coefficients associated with the respondents’ preferences varying randomly over the respondents, and *x_in_* is a vector of the product attributes for individual *n* and alternative *i* in the choice set.

The data were modeled using the latent class model (**LCM**), which takes the heterogeneity of respondents’ preferences into account by dividing respondents into classes and estimating a choice model for each of them (Louviere et al., 2000; Hensher et al., 2015). The LCM is an extension of the conditional logit model (see McFadden, 1974), which represents the average preference of the respondents and assumes similar preferences for them, and that random error terms follow the Type 1 extreme value distribution and are independently and identically distributed across alternatives (Greene and Hensher, 2003). Each “latent” class has a unique marginal utility. Compared to the conditional logit model, independent distributions are not required by the LCM (Boxall and Adamowicz, 2002; Train, 2003).

Choice situations present alternatives with varying attribute levels. The possibility of respondent *n* choosing alternative *i* is given by the probability that the utility gained from alternative *i* is equal to or greater than the utility gained from the set of alternatives *j* in the choice set. This is presented as follows:

$$P_{in}=P\left[\left(V_{in}+\varepsilon_{in}\right)\geq P\left(V_{jn}+\varepsilon_{jn}\right)\;\forall j;i\neq j\right]$$

where *P_in_* is the likelihood of respondent *n* choosing alternative *i*. A no-choice option was also presented to the participants, because this follows ordinary shopping behavior (Adamowicz et al., 1998). Alternative specific constants for each alternative (A, B, C) were also included in the model to capture the systematic bias of choosing the same option over another (e.g., possible tendency of selecting the option on the left), which capture the variation in choices that is not explained by the attributes.

The probability that individual *n* belongs to latent class *s* and will select alternative *i* can be written as:

$$P_{ins}=\sum_{s=1}^{s}\left[\frac{e^{\alpha \lambda_{s}z_{n}}}{\sum_{s=1}^{s}e^{\alpha \lambda_{s}z_{n}}}\right]*\left[\frac{e^{\mu_{S}\beta_{S}X_{i}}}{\sum_{j=1}^{J}e^{\mu_{S}\beta_{S}X_{j}}}\right],$$

where *x* is a vector of attributes of an alternative, *β_s_* is a segment-specific utility, *μ_s_* is a scale parameter, and *Z_n_* denotes the covariates, for example, in this study trust, opinion on the finance system and sociodemographic characteristics of the consumers. The groups were determined based on the respondents’ preferences of FAW attributes and price level choices, so covariates did not affect the latent class model, being inactive (Appendix 2), as the target is to examine the WTP segments for various AW measures. The price was treated as a continuous variable in the modeling. To allow the easy interpretation of enhanced FAW, the improved level of FAW was compared with the current (the first, or base) level. The number of classes was determined based on several criteria, that is, Akaike's (**AIC**), corrected AIC (**CAIC**), and Bayesian (**BIC**) (Train, 2003, Appendix 3).

The estimated model coefficients were not interpretable in economic terms, because discrete choice models measured the utility of the respondents. The implicit price estimates of the FAW attributes were calculated as the quotient of the coefficient of attribute (*k*th) and the price coefficient (*p*) to estimate the WTP values:

$$\text{WT}P_{k}=-\frac{\beta_{k}}{\beta_{p}}.$$

Latent Gold 5.1 (Statistical Innovation Inc.) was used to model the data for latent classes.

### Logistic Regression

After estimating the most likely coherent consumer groups by using the latent class model, the individual characteristics of the latent groups were examined. Logistic regression models were estimated to explain the characteristics of each respondent group estimated by the LCM (see, e.g., Greene, 2008). Logistic regression is a binary choice model, which is typically estimated with the maximum likelihood method, where the dependent variable takes either the value 0 or the value 1. Each respondent class resulting from the latent class modeling was compared (one at a time) with the other classes (e.g., Greene, 2008):

$$P_{n}\left(Y_{n}=1|X_{n}\right)=\frac{e^{\beta x_{n}}}{1+e^{\beta x_{n}}},$$

where *Y* is the binary variable outcome (1 or 0), *P* is the probability of *Y*, and *x* are the independent variables. Explanatory variables included in the model were selected so that the specifications with the best goodness of fit for the model were selected. The vector of the explanatory variables $x_{n}\;$included several variables describing the respondents’ opinions on finance and information sources and socioeconomic status. The relevance and the respondent's connection with and knowledge of animal production as explanatory variables were also tested.

Before selecting the logistic regression model, also other distribution specifications were tested. The use of logistic regression was supported by the high probabilities of respondents to belong or not to belong to the group (shown in the Appendix 4). With lower probabilities than those obtained, other distribution assumptions than logistic model would have been relevant. With the current the linear regression model and log(1 + *y*) transformation for probabilities, the assumption regarding residuals would have been violated.

Several consumer groups were identified in the analysis. The variables used in the latent class analysis are presented in Table 2. Each model explained the likelihood of a respondent belonging to a certain group. Statistically insignificant explanatory variables were excluded from the estimated model by using backward elimination. In each iteration, the least significant variable was dropped until all the remaining variables were statistically significant at a 5% risk level. A similar procedure was repeated for all 5 groups identified in the latent class analysis.

**Table 2:** Share of respondents, who considered certain financing approach for an FAW labeling scheme as a good suggestion, and opinions on trust toward FAW information sources, development of FAW and connections with or knowledge of animal production, and sociodemographic characteristics.

Variable	Share of respondents %	Finnish population % (2018)
Female^1^	49	51
Residence^2^: Helsinki metropolitan area	21	21
Urban municipality (>15k inhabitants)	58	58
Periurban or rural municipality	22	21
Age group^1^ (years): 18–24	10	10
25–34	14	16
35–44	16	15
45–54	18	15
Over 55	42	44
Income group^3^ (annual): <€20,000	29	40
€20,001–€30,000	11	20
€30,001–€40,000	22	17
€40,001–€60 000	13	15
>€60,000	3	8
Income data not available	22	
Education^4^: Basic	9	27
Upper secondary education	36	41
College degree/other	16	10
Bachelor's degree	22	12
Master's/Doctor's degree	18	11
Suggested method of financing an animal welfare labeling scheme considered as a good suggestion:		
Consumers pay premium. The profit is passed on to the producers by the retail company	67	
Consumers pay premium. The profit is shared by the food chain's actors based on the actual costs incurred	49	
Retail and food industry finance a fund, which covers the cost for the actors	55	
The government pays for producers from tax funds	44	
Increased VAT in AW products, which is allocated to producers in the system	37	
Suggested animal welfare information source considered trusted		
Produced in Finland—organization	66	
Authorities	60	
Livestock producers	51	
Animal protection organization	50	
Retail companies and organizations	38	
Established company for managing FAW label system	32	
Food processing companies	31	
Knowledge of and connections with farms		
Considers themselves to know (quite) a lot about animal production	17	
No connections with animal farms	45	
Lived on production farms	14	

1 Data on Finland's population: Statistics Finland. 11ra—Key figures on population by region, 1990 to 2021.

2 Data on Finland's population: Statistics Finland. 12au—Vital statistics and population by area, 1990 to 2021.

3 Data on Finland's population: Vero.fi Statistical databases. 2. Individual taxpayers—Statistics on income taxation. Underage persons excluded.

4 Data on Finland's population: Statistics Finland—Educational structure of population for the 15 yr and older.

### Explanatory Variables and Regression Modeling

Sociodemographic variables (e.g., age, income, highest education obtained, type of area where the respondent lived, gender), variables concerning familiarity, and connections with the production farms (living/having lived on a farm) were included in the estimation.

The majority of respondents were in living urban municipalities (minimum 15,000 inhabitants) rather than in the Helsinki metropolitan or periurban and rural areas. The median annual income per adult in the household was between €20,000 and €30,000. However, income information was not provided by 22% of respondents. The gender, type of residence and age in the sample represented Finnish population well. Regarding education, the respondents in the sample were somewhat more educated than the residents of Finland on average. Only 17% of respondents considered their (subjective) knowledge of animal production (quite) good, 45% of the respondents had no connections to farm animals whereas 14% of the respondents currently lived or had lived at a farm.

Trust in actors was measured by questions that assessed the reliability of different actors as an information source regarding a FAW label. The dummy variable coding “trusted” referred to the responses “reliable” or “very reliable” in the 5-point Likert scale used in the original question. The following food chain actors were included, because they could be relevant actors in establishing a FAW label system: Produced in Finland—label organization, a company established for a FAW label, livestock producers; retail groups; authorities; and animal protection organizations. Retail was excluded from the list of actors because of multicollinearity issues when including both food processing and retailers. The authorities can play an important role in controlling and regulating the animal production sector, but animal protection parties can also play an important role as mediators and social influencers, and in some countries, they have established FAW assurance schemes. While people's attitudes toward animal protection organizations differ due to opinions concerning animal welfare violations and the acts or campaigns of some actors, this can be a useful proxy for people's attitudes. The authorities, livestock producers, and animal protection organizations were considered trusted sources of information by 60, 51, and 50% of respondents, respectively.

The respondents were asked for their opinion regarding the financial responsibilities borne by various actors and proposed models for obtaining funding that covers the costs of an animal welfare label. The consumers were asked to assess on a 5-point scale whether they considered each proposition a poor or good suggestion. Several actors and models for bearing financial responsibility were proposed, including that the costs of animal welfare labeling were beared by i) taxpayers, ii) an industry fund, iii) increased VAT in FAW labeled products, and iv/v) consumers through a price premium. A premium paid directly by the consumers was presented as 2 individual options, where iv) the premium was transferred to the producers only or v) to all the food chain actors based on their actual incurred costs. Both the premium option iv) and industry fund ii) option were considered a good alternative by the majority of respondents (67 and 55% of respondents, respectively, Table 2), but the iii) higher VAT only by 37% of the respondents. Livestock producers bear not only responsibility for FAW, but possibly also the financial burden from FAW measures due to more administrative costs, inspection, and investment, which can be acknowledged and valued varyingly and affiliated with trust in producers’ actions with animals. Especially i) represented a more indirect approach than consumer-paid premiums. However, with the taxes, the financial responsibility for the scheme would fall on society and eventually, citizens.

## RESULTS

### Latent Class Estimation Results

Several LCMs were estimated. Based on several criteria, including Akaike's (AIC), corrected AIC CAIC, and Bayesian (BIC) information criteria, the 5-group model was considered most suitable with the lowest BIC value and low CAIC value (Appendix 3). The model of 3 (or 4) classes could have been considered based on the change in the values, but the model of 5 classes provides the best explanatory power for the groups. The conditional logit model (**CLM**) with one class illustrated the overall view of consumer preferences for the studied FAW characteristics of broiler fillet. The *R*² value of the final LCM (Table 3) was substantially higher (0.41) than the *R*^2^ value of the CLM (0.09). Hence, the results supported the choice of the latent class model of 5 classes rather than the CLM. Table 3 represents the coefficients for each attribute and for the alternative-specific constants, including the opt-out (“no buy”) option (an alternative-specific constant). Based on the results, none of the options were not prioritized (representing selecting the same option regardless of the content). The first levels of categorical variables were used as a reference level. The estimates for the CLM followed the hypothesis that the more ambitious the welfare measure, the larger the coefficient. For example, the stocking density of 14 to 15 birds per m^2^, had a larger positive coefficient than the stocking density of 16 to 17 birds per m^2^, which is the stocking density that is currently common. The price coefficient was negative as expected, suggesting that the higher the price, the lower the likelihood the product will be chosen.

**Table 3:** Results of the conditional logit model (CLM) and latent class model (LCM) of 5 groups, showing the likelihood of a survey respondent selecting a product with a given set of attributes.

	Conditional logit	Latent class						
Group name and number	Overall	1) Main group, average WTP, FAW supporters	2) Minor WTP, young	3) Heavy supporters, FAW product byers	4) FAW focused, high WTP	5) Criticals, basic need focused	LCM overall	
*R*²	0.08	0.14	0.52	0.33	0.16	0.08	0.41	
*R*² (0)	0.09	0.22	0.53	0.40	0.19	0.89	0.41	
Class size, % respondents	100%	41%	19%	17%	12%	11%		
Attributes							*P* value	Wald *P* value
Alternative-specific constant 1							<0.001	<0.001
Alternative-specific constant 2	0.144**	0.054	0.478	0.080	0.131	0.750		
Alternative-specific constant 3	−0.119*	−0.415***	1.214***	−0.311*	−0.410	0.130		
Alternative-specific constant 4 (opt-out)	−0.75***	−2.847***	−2.491***	−4.926	0.760**	2.020		
Stocking density: 18 birds per m²							<0.001	<0.001
16–17 birds per m²	0.515***	0.240**	1.206***	0.563**	1.213***	1.732*		
15 birds per m²	0.784***	0.481***	1.727***	1.527***	1.700***	1.897		
Enrichments: No requirements							<0.001	<0.15
Ramps and levels	0.578***	0.465***	0.583***	0.994***	1.471***	0.638		
Ramps, levels, and dust bathing	0.783***	0.744***	0.890***	1.400***	1.563***	1.329		
Litter quality: No requirements							<0.001	<0.001
Monitor and action plan	0.464***	0.208**	1.629***	0.790***	0.661**	2.365**		
Foot health: No requirements							<0.001	<0.001
Monitor and action plan	0.413***	−0.001	1.515***	1.04***	1.40***	1.958**		
Price	−0.001***	−0.002***	−0.010***	0.001***	−0.001**	−0.019*	<0.001	<0.001

Statistical significance level: *** 99%, ** 95%, * 90%.

When evaluating the results of the LCM, a negative price coefficient was obtained for groups 1, 2, 4, and 5, as presented in Table 3. Groups were named based on the results of the LCM and logistic regression analysis. The Wald *P* value was statistically insignificant for the provision of enrichments attribute, which implied that it was class-independent. The first group, called as the *main group, with average WTP, FAW supporters*, included 41% of the respondents in the sample. Apart from the statistically insignificant attribute of foot health surveillance, all the attributes again followed the hypothesis that the higher the level of welfare, the larger the coefficient. The second group included 19% of the respondents and was called *the minor WTP, young- group*. Welfare attributes’ coefficients were statistically significant at a risk level of 5% or lower, and the parameters were logical.

In the third group, which included 17% of the respondents, a positive price coefficient was obtained. Hence, the estimate was counter to expectations, because the theory would require a negative price coefficient. The results also indicated that “*heavy supporters*,” FAW *product buyers’-group* had always chosen one of the offered products and higher price levels in each choice situation (Appendix 5A and 5B). The fourth group included 12% of respondents, and it was called the *FAW focused group with high WTP*. The parameter estimates for all the welfare attributes were significant and logical, as for the other groups above. Group 5 included only 11% of respondents and had statistically significant parameter estimates only for the first additional level of stocking density (16–17 birds per m^2^), litter quality monitoring and foot health surveillance. Based on the results, the respondents belonging to this group appreciated only the basic needs of animals, such as good health and hence it was named as *the critical, basic need focused-group*.

### Willingness to Pay Estimates

The price premiums for different welfare attributes were derived from the LCM results are presented in Table 4. WTP was not calculated for statistically insignificant parameters. In addition, a positive price parameter prevented the determination of valid positive implicit prices for the *Heavy supporters, FAW product buyers*’ group for which the coefficients were positive. However, the group avoided the opt-out-option.

**Table 4:** WTP an additional price premium for FAW attributes per consumer group in 4 consumer groups, euros per attribute for 400 g of broiler chicken fillet.

Attribute levels	1) Main group, average WTP, FAW supporters	2) Minor WTP, young	4) FAW focused, high WTP	5) Criticals, basic need focused
Group size, % of respondents	41%	19%	12%	11%
Stocking density—More space, 16–17 birds per m²	1.42	1.22	17.39	0.93
Even more space, 14–15 birds per m²	2.85	1.74	24.37	
Enrichments: Ramps and levels	2.76	0.59	21.10	
Enrichments: Ramps, levels and dust bathing	4.40	0.90	22.41	
Litter: Monitoring and improvements	1.23	1.64	9.47	1.27
Foot health: Monitoring and improvements		1.53	20.10	1.05

The *main group, FAW supporters with an average WTP*, was willing to pay on average a price premium of €1.42 to €2.85 for additional space, €2.74 to €4.40 for enrichments, and €1.23 for litter enhanced quality, but nothing for foot health surveillance. These premiums were in addition to the base price of €4.00 for a 400 g package of broiler fillet. The *price conscious, young people-group* had a positive WTP for every additional welfare level, but the premium was smaller than in *the main group*. In this group, the highest WTP estimates were obtained for the highest level of stocking density, litter quality, and foot health surveillance (€1.74, €1.64, and €1.53, respectively). The WTP for enrichments provision, ramps, and levels was only €0.59. The FAW *focused group with a high WTP* showed a very high WTP, ranging up to €24.37, suggesting that the price was a secondary factor in this group. Group 5, being *the criticals* and focusing on the basic needs, was only slightly interested in FAW and willing to pay a premium (€0.93–€1.27 per attribute) only for the level of a maximum of 16 to 17 birds per m^2^ of stocking density, enhanced litter quality, and foot health surveillance, but was unwilling to pay a premium for additional enrichments.

WTP for enrichments provision and foot health monitoring varied greatly between the groups, whereas improvements in stocking density and litter quality were preferred in every group. The results for enrichments were interesting: Compared with other attributes, the strongest preference for enrichments existed in the *main group* and *FAW focused group with high WTP*, but the lowest preference existed in the *Minor WTP, young-group*, and no WTP in the *critical, basic need focused group* was observed.

If all the welfare measures for which the main group was WTP were calculated by the group's WTP values, the additional price premium was €5.41, resulting in a total of €9.41 per 400 g package for a stocking density of 16 to 17 birds per m^2^, litter monitoring, and ramps and levels. Based on the results, when all 4 FAW measures were taken together, the total price premium plus the base price for the *price conscious* group and *basic needs focused* group was €13.00 and €11.24 per 400 g meat, respectively. For comparison, in April 2021, the price of conventional unseasoned boneless chicken fillet in 2 Finnish supermarkets ranged from €2.36 to €6.74 per 400 g, and there were also more expensive seasoned products on the market. However, the price for 400 g of organic broiler chicken fillet was approximately €12 or more.

### Logistic Regression Analysis

Logistic regression analysis was conducted to identify possible differences between groups’ characteristics. The analysis was run individually for each consumer group, using the other respondents as a reference. The Nagelkerke values varied between 0.059 and 0.284 in the groups. The Hosmer-Lemeshow goodness-of-fit values were higher than 0.05 for all 5 groups.

Regarding the sociodemographic variables, the respondent's age, and connections with livestock farms were the variables which had the strongest association with the likelihood of a respondent to belong to a certain group. The probability of a respondent belonging to the *main group with average WTP* increased when the respondent was a male aged between 35 and 44 or over 55, compared to the reference age class of 18 to 24 (Table 5). The respondents who considered the proposition of financing an AW label scheme through tax funds a good idea, but had a tendency (0.05 < *P* < 0.1) for not favoring consumers paying increased value-added tax allocated to producers and those who did not trust the nongovernmental animal protection organizations as the source of FAW information were more likely to belong to the main group than their counterparts.

**Table 5:** The results from the logistic regression profiling the 5 consumer classes.

Attribute levels	1) Main group. average WTP, FAW supporters	2) Minor WTP, young price conscious	3) Heavy supporters, FAW product byers	4) FAW focused. high WTP	5) Criticals, basic need focused
	Regression coefficient (standard error) and the significance level *** 99%. **95%. *90%				
Female	−0.798 (0.312)***				
Age group: 18–24 yr					
25–34 yr	0.535 (0.467)	−0.906 (0.458)**			1.415 (0.784)*
35–44 yr	1.739 (0.469)***	−1.313 (0.471)***			−1.091 (1.24)
45–54 yr	0.069 (0.467)	−0.898 (0.428)**			1.261 (0.775)
over 55 yr	0.876 (0.413)**	−1.321 (0.382)***			1.311 (0.742)*
Education: Basic					
Upper secondary education	0.617 (0.43)				1.086 (0.807)
College degree/other*	0.607 (0.474)				−0.527 (0.987)
Bachelor's degree	0.879 (0.453)*				0.22 (0.878)
Master's/Doctor's degree	−0.098 (0.468)				1.17 (0.84)
Lived or have lived at the farm			0.883 (0.342)***		
No connection to production animals				0.753 (0.342)**	
No consumption of red meat					1.682 (0.661)**
Finance system: Tax fund for producers	0.564 (0.246)**				−0.788 (0.404)*
Price premium allocated to producers					−1.18 (0.362)***
Price premium allocated to food chain actors			0.596 (0.296)**	−0.57 (0.349)	
Increased VAT in FAW products allocated to producers	−0.528 (0.244)**		0.533 (0.288)*		
Trust: Animal protection	−0.556 (0.24)**		0.77 (0.299)***	1.121 (0.37)***	
Authorities		0.456 (0.27)*	−0.727 (0.304)**		
Animal producers			0.545 (0.294)*	−0.591 (0.347)*	
Processing industry					−1.534 (0.559)***
Good from Finland—organization	0.408 (0.249)				
Constant	−1.312 (0.563)**	−0.639 (0.365)*	−2.609 (0.334)***	−2.678 (0.388)***	−2.665***
Chi-square	51.702	15.411	33.312	18.408	62.668
Nagelkerke *R*^2^	0.164	0.059	0.133	0.092	0.284
Hosmer and Lemeshow *P* value	0.885	0.286	0.289	0.399	0.918

There was a reduced likelihood of belonging to the *minor WTP, young group* among respondents in any age group from 25 yr to over 55 yr. Respondents, who trusted the authorities as source of AW information were more likely to belong (a tendency 0.05 < *P* < 0.1) to the *minor WTP, young- group* than their counterparts. Therefore, the young age defined the group the most.

There was an elevated likelihood of a respondent belonging to the *heavy supporters, AW product buyer* group among respondents who currently lived or had previously lived on a farm, who trusted animal protection organizations and also producers (a tendency), but a reduced likelihood for those who did trust the authorities as the source of FAW information. In addition, there was of an elevated likelihood of a respondent belonging to the *heavy supporters*, FAW *product buyer* group among those respondents who considered the funding mechanism which allocated the price premium to food chain actors and a tendency (0.05 ≤ *P* ≤ 0.1) for considering increased VAT in FAW products allocated to producers as good idea.

There was an elevated likelihood of a respondent belonging to the FAW *focused* group with high WTP among those who had no connections with animal production or trusted animal protection organizations as the source of FAW information. In addition, there was a tendency (0.05 ≤ *P* ≤ 0.1) of a reduced likelihood of a respondent belonging to the *FAW focused* group with high WTP among those who trusted animal producers as the source of FAW information. There was an elevated likelihood of a respondent belonging to *the critical, basic need focused* group among those aged between 25 and 34, and respondents aged of 55 and older. An elevated likelihood was also associated with the respondent not consuming red meat. By contrast, there was a reduced likelihood of a respondent to belong to *the critical, basic need focused* group when s/he considered only the funding of a FAW labeling scheme through a price premium allocated to livestock producers a good idea or price allocated to the food chain actors. Finally, respondents who did not trust the processing industry as the source of FAW information were less likely to belong to this group.

## DISCUSSION

The results suggested that Finnish consumers in general have a positive WTP for improvements in FAW in broiler production, but some consumer segments which differ by their WTP exist. In the current study, 5 different groups were identified. The preferred welfare measures, if WTP is used to indicate the preference, were additional space allowance, with a level of 16 to 17 birds per m^2^ and enhanced litter quality monitoring at the farm, which both affect, for example, broiler health (Dawkins et al., 2004; Allain et al., 2009). The WTP for the provision of enrichments substantially differed by group. Based on the results, *the main* group supporting FAW, *FAW focused* group with high WTP and *the heavy supporters, FAW product buyers group* (based on the coefficients) appreciated it most (in relative terms), whereas the *Minor WTP, young group*, and *the critical, basic need focused-group* valued the animal health-related attributes more than enrichments. The provision of enrichments provides more possibilities to express animals’ natural behaviors.

The results support the results by Clark et al. (2016) that the consumers’ recognition of FAW has grown beyond health and housing, extending to, for example, natural behavior. The positive attitude toward behavior-related attributes signals more WTP for FAW than only basic needs such as health.

For some attributes, the WTP estimates obtained in the current study were fairly high compared with the results reported in some of the previous studies (e.g., Cicia and Colantuoni, 2010; Clark et al., 2017), but for individual attributes, the final product price was nevertheless feasible compared with current chicken prices in Finland. In the current market situation, the supply of organic chicken meat in Finland and most other countries in Europe is low (Willer et al., 2021). However, “mid-market” production has started to gain market share in some countries and it can produce welfare cost-effectively (see, e.g., Gocsik et al., 2016). Hence, animal-friendly labeled products could increase the choice of products in grocery stores and meet the expectations of consumers who are unwilling to pay enough for organic chicken, but nevertheless would like to have products with an enhanced level of animal welfare. In this respect, the results support the view of Denver et al. (2017) in that there may be room for mid-market products with a more competitive price than organic products, and that still provide additional value to many consumers by improving animal welfare in some key areas of welfare, but not necessarily in all areas.

The processes of mass broiler production are scrutinized by the broader public more and more (Bos et al., 2018). Consumer trust in food system actors and the information they provide, and the sense of responsibility are of vital importance when introducing premium labeled products to the market. The results suggest that trust in various actors and who is expected to bear financial responsibility for animal welfare influence consumer WTP for animal welfare improvements. Although the interest in buying FAW products seems common between the groups (*heavy supporters, FAW product buyers-group, and FAW focused group with high WTP*), the attitude toward animal producers and connections with animal farming diverged. Closer connections with and trust in producers (i.e., a respondent living or who had lived on a farm and trust in producers (and animal protection organizations)) as a source of information characterized the *heavy supporters, FAW buyers* group, whereas no connections with farming, a strong trust in animal protection organizations, and mistrust in producers as providers of FAW information defined the *FAW focused with high WTP* group.

We expected that the positive price coefficient of the *FAW product buyers group* resulted in the choosing of higher price levels, which may have indicated quality in this case, so the higher price indicated higher welfare, as discussed, for example, by McVittie et al. (2006). The *FAW focused with high WTP* group had very high WTP values. This suggests that consumers’ attitude toward producers can be an essential factor that challenges, for example, the control system of FAW for being reliable and in gaining trust among the *FAW focused with high WTP* group. This suggests that partnering with animal protection organizations and engaging consumers in interaction can be an effective labeling strategy in this consumer segment. Connections with production farms, as associated with the FAW product buyers, have been discussed in the previous studies in relation to knowledge, and the information about agricultural practices may increase the WTP and valuation of FAW (Umberger et al., 2009; Gwin et al., 2012), and the need for concerted and interactive communication with the public has been suggested (Clark et al., 2019).

The results suggest that the *main group* was characterized by a mistrust in animal protection organizations as providers of FAW information. The taxpayer-funded approach was preferred by the *main group*, which suggests that it was felt that there should be shared responsibility for FAW improvements. This may also suggest a preference for a more indirect system, in which the actual consumer of a labeled product does not bear much financial responsibility. Overall, these aspects suggest that the *main group* somehow externalizes the responsibility for FAW improvements to other actors in society and prefers the citizens (nonconsumers) involvement. Therefore, for this consumer segment, a public policy-oriented approach to improving animal welfare can be recommended, and the size of this group highlights the need for agricultural policies such as animal welfare compensation and the Common Agricultural Policy to support animal welfare improvements. In this respect, an important task for both national policies and the European Union's Farm to Fork strategy (European Commission, 2020) is to advance FAW improvements by means of both policies and market-based measures such as FAW labeling. Previous studies have also suggested collaborative efforts between policy and business (Niemi et al., 2020).

Younger age determined *the minor WTP-group* but also the *criticals*, as a young age (18–24 yr) increased the likelihood of belonging to the *minor WTP, young-group* and age group of 25 to 34 had increased tendency of belonging to the *critical, basic needs focused- group*. These groups are also likely to be those who would be satisfied with smaller FAW improvements and mid-market products, as long as they are priced competitively. Additionally, the results suggested that the *critical, basic need focused-group* considered neither consumer paid premiums for producers nor the whole food chain a good suggestion, and appeared to consume less red meat. The negative attitude may be a sign of disinterest in FAW in general, which is also in line with the modest WTP values considering only the basic needs. However, the disinterest may also be related to the avoidance of animal-sourced food, which is associated with the citizen role and being critical of FAW (Clark et al., 2016). The group may therefore be polarized.

The current approach may have some limitations that need to be kept in mind when interpreting the results. The Choice Experiment as an ex ante valuation method may overestimate the WTP and may lead to a bias (Hausmann, 2012), explained, for example, by people deriving utility from a positive self-image associated with morally commendable behavior (Johansson-Stenman and Svedsäter, 2012). If such actions are free of charge at the time of making the choice representing social desirability bias, which is linked to animal welfare (Norwood and Lusk, 2011). Ex ante approaches, such cheap talk script, have been found successful (e.g., Champ et al., 2009) in enhancing the data, while others observed fewer effect (Bosworth and Taylor, 2012). Additionally, given that we examined a new product signaling (presented, e.g., Lusk et al., 2007; Doyon and Bergeron, 2016) encouraging the provision of new product could be an appropriate explanation for rather high WTP. Furthermore, the information given in the survey in the definitions of welfare measures may be linked with high WTP values as the knowledge concerning production practices increases (Lusk, 2018). There was plenty of information of the FAW practices in the survey, for the needs of enabling the respondent to weight the AW attributes. Moreover, the attributes were limited to only FAW to the study needs and to not make the choices too hard on respondents, even though in the real purchase situation also other attributes such as environmental friendly, brand, appearance and healthiness, may be evaluated by the consumer. Moreover, although the sample was fairly small, the results were logical and the main effects were clear.

In conclusion, there is demand for animal-friendly labeled broiler chicken among Finnish consumers. However, consumer expectations are heterogeneous, and at least 4 different strategies can appeal to different segments. While one consumer segment prioritizes FAW over price, another segment emphasizes low-priced products and considers mid-market products appealing. Moreover, public policies are an important tool for enhancing FAW for a large number of consumers, and public actions are therefore warranted. Finally, engaging with animal protection organizations (as mediators) and being open to consumers can be an effective strategy for building confidence in premium products.

## Appendix Supplementary materials

Image, application 1

## Footnotes

Supplementary material associated with this article can be found in the online version at doi:10.1016/j.psj.2023.102765.
