# Supplementary material for: Consumer trust and willingness to pay for establishing a market-based animal welfare assurance scheme for broiler chickens
Source: Poult Sci. 2023 Apr 29;102(7):102765. doi: 10.1016/j.psj.2023.102765 (PMC10227368; doi:10.1016/j.psj.2023.102765)
Supplement: Supplementary file 1 [file mmc1.docx]

Appendix 1. The importance of certain FAW criteria to 400 respondents “How important do you consider the following criteria to be included in the FAW label?”

Description of the procedures

*Monitoring of foot health preventively at the farm*: Lameness of broilers and the condition of their feet (foot pad, hock) would be systematically monitored on broiler farms. Foot health is essential to broiler health and welfare.

*Appropriate treatment and handling - training for animal keepers*: Animal keepers would be trained to take into account the species-specific behavior of animals and support it with appropriate practices

*Provision of enrichments*: Broilers would be provided with dust bathing boxes with dry bedding for bathing. The use of dust bathing boxes enables the specie-spesific behavior

*Use of elevated grids:* The broiler house would be equipped with elevated grids on which the birds can climb. The use of grids increases the broiler's possibilities to move.

*A comfortable bedding:* The quality of the soft material added to the floor would be controlled according to a pre-prepared plan. Too damp and dirty bedding predisposes broilers to foot pad lesions

*Preventive health care:* A farm-specific plan would be drawn up for the prevention of illnesses and injuries. Prevention is aimed at healthy animals.

*More living space:* There should be no more than 33 kg (about 14-15 birds) of broilers per square meter. Nowadays, there can be no more than 42 kg (about 18 birds) of birds per square meter. It is easier for broilers to move when they have more space available.

*Slower growing breed:* A slower growing broiler breed would be chosen for production. In normal production, broilers grow for 4-6 weeks. For example, in organic production, slower growing breeds are used, when the production time of the birds is 8- 9weeks.

Appendix 2. Inactive covariate profiles and probability means.

| Covariates PROFILE | |  |  |  |  | Covariates PROBMEANS | |  |  |  |  |
| --- | --- | --- | --- | --- | --- | --- | --- | --- | --- | --- | --- |
|  | Class1 | Class2 | Class3 | Class4 | Class5 |  | Class1 | Class2 | Class3 | Class4 | Class5 |
| Male | 0.59 | 0.48 | 0.45 | 0.37 | 0.51 | Male | 0.48 | 0.18 | 0.15 | 0.08 | 0.12 |
| Female | 0.41 | 0.52 | 0.55 | 0.63 | 0.49 | Female | 0.35 | 0.20 | 0.19 | 0.15 | 0.11 |
| *Residence*: Helsinki metropolitan area | | | |  |  | *Residence*: Helsinki metropolitan area | | | |  |  |
| 0 | 0.78 | 0.83 | 0.87 | 0.71 | 0.79 | 0 | 0.41 | 0.20 | 0.19 | 0.11 | 0.10 |
| 1 | 0.22 | 0.17 | 0.13 | 0.29 | 0.21 | 1 | 0.43 | 0.15 | 0.11 | 0.16 | 0.15 |
| Urban municipality (>15k inhabitants) | | | |  |  | Urban municipality (>15k inhabitants) | | | |  |  |
| 0 | 0.42 | 0.35 | 0.44 | 0.52 | 0.43 | 0 | 0.41 | 0.15 | 0.18 | 0.14 | 0.12 |
| 1 | 0.58 | 0.65 | 0.56 | 0.48 | 0.58 | 1 | 0.42 | 0.21 | 0.17 | 0.10 | 0.11 |
| Peri-urban or rural municipality | | | |  |  | Peri-urban or rural municipality | | | |  |  |
| 0 | 0.79 | 0.82 | 0.69 | 0.77 | 0.78 | 0 | 0.42 | 0.20 | 0.15 | 0.12 | 0.12 |
| 1 | 0.21 | 0.18 | 0.31 | 0.23 | 0.22 | 1 | 0.39 | 0.15 | 0.24 | 0.12 | 0.09 |
| *Age group:* 18–24 years | | | | | | *Age group***:** 18–24 years | | | | | |
| 0 | 0.92 | 0.82 | 0.91 | 0.90 | 0.90 | 0 | 0.42 | 0.17 | 0.17 | 0.12 | 0.12 |
| 1 | 0.08 | 0.18 | 0.09 | 0.10 | 0.10 | 1 | 0.34 | 0.33 | 0.15 | 0.11 | 0.07 |
| Age group: 25–34 years | | | | | | Age group: 25–34 years | | | | | |
| 0 | 0.86 | 0.84 | 0.90 | 0.90 | 0.86 | 0 | 0.41 | 0.18 | 0.18 | 0.12 | 0.10 |
| 1 | 0.14 | 0.16 | 0.10 | 0.10 | 0.14 | 1 | 0.42 | 0.20 | 0.12 | 0.08 | 0.17 |
| Age group: 35–44 years | | | | | | Age group: 35–44 years | | | | | |
| 0 | 0.79 | 0.86 | 0.89 | 0.82 | 0.85 | 0 | 0.39 | 0.19 | 0.18 | 0.11 | 0.13 |
| 1 | 0.21 | 0.14 | 0.11 | 0.18 | 0.15 | 1 | 0.56 | 0.17 | 0.12 | 0.13 | 0.02 |
| Age group:45–54 years | | | | | | Age group 45–54 years | | | | | |
| 0 | 0.86 | 0.82 | 0.79 | 0.78 | 0.82 | 0 | 0.43 | 0.19 | 0.16 | 0.11 | 0.11 |
| 1 | 0.14 | 0.18 | 0.21 | 0.22 | 0.18 | 1 | 0.32 | 0.19 | 0.21 | 0.15 | 0.14 |
| Age group: over 54 years | | | | | | Age group: over 54 years | | | | | |
| 0 | 0.57 | 0.66 | 0.52 | 0.60 | 0.58 | 0 | 0.41 | 0.21 | 0.15 | 0.12 | 0.10 |
| 1 | 0.43 | 0.34 | 0.48 | 0.40 | 0.42 | 1 | 0.42 | 0.15 | 0.20 | 0.11 | 0.13 |
| Education: Basic | |  |  |  |  | Education: Basic | |  |  |  |  |
| 0 | 0.93 | 0.90 | 0.88 | 0.88 | 0.91 | 0 | 0.42 | 0.18 | 0.17 | 0.11 | 0.12 |
| 1 | 0.07 | 0.10 | 0.12 | 0.12 | 0.09 | 1 | 0.34 | 0.22 | 0.23 | 0.16 | 0.06 |
| College degree/other* | | |  |  |  | College degree/other* | | |  |  |  |
| 0 | 0.83 | 0.82 | 0.83 | 0.85 | 0.84 | 0 | 0.41 | 0.18 | 0.17 | 0.12 | 0.13 |
| 1 | 0.17 | 0.18 | 0.17 | 0.15 | 0.16 | 1 | 0.45 | 0.21 | 0.18 | 0.11 | 0.05 |
| Upper secondary education | | | | | | Upper secondary education | | | | | |
| 0 | 0.65 | 0.66 | 0.69 | 0.60 | 0.64 | 0 | 0.42 | 0.19 | 0.18 | 0.11 | 0.09 |
| 1 | 0.35 | 0.34 | 0.31 | 0.40 | 0.36 | 1 | 0.40 | 0.18 | 0.15 | 0.13 | 0.15 |
| Bachelor’s degree | | | | | | Bachelor’s degree | | | | | |
| 0 | 0.74 | 0.82 | 0.78 | 0.82 | 0.79 | 0 | 0.39 | 0.19 | 0.17 | 0.12 | 0.12 |
| 1 | 0.26 | 0.18 | 0.22 | 0.18 | 0.22 | 1 | 0.49 | 0.15 | 0.17 | 0.10 | 0.08 |
| Master’s/Doctor’s degree | | | | | | Master’s/Doctor’s degree | | | | | |
| 0 | 0.74 | 0.82 | 0.78 | 0.82 | 0.79 | 0 | 0.39 | 0.19 | 0.17 | 0.12 | 0.12 |
| 1 | 0.26 | 0.18 | 0.22 | 0.18 | 0.22 | 1 | 0.49 | 0.15 | 0.17 | 0.10 | 0.08 |
| *Method of financing considered a good suggestion***:** Consumers pay premium, which is shared by the food chain’s actors | | | | | | *Method of financing considered a good suggestion***:** Consumers pay premium, which is shared by the food chain’s actors | | | | | |
| 0 | 0.34 | 0.28 | 0.22 | 0.32 | 0.34 | 0 | 0.42 | 0.16 | 0.11 | 0.11 | 0.20 |
| 1 | 0.66 | 0.72 | 0.78 | 0.68 | 0.67 | 1 | 0.41 | 0.20 | 0.20 | 0.12 | 0.07 |
| Consumers pay premium, which is passed on to the producers | | | | | | Consumers pay premium, which is passed on to the producers | | | | | |
| 0 | 0.51 | 0.48 | 0.35 | 0.60 | 0.51 | 0 | 0.41 | 0.17 | 0.12 | 0.14 | 0.16 |
| 1 | 0.49 | 0.52 | 0.65 | 0.41 | 0.49 | 1 | 0.41 | 0.20 | 0.23 | 0.10 | 0.07 |
| Retail and food industry finance a fund, which covers the cost for the actors | | | | | | Retail and food industry finance a fund, which covers the cost for the actors | | | | | |
| 0 | 0.44 | 0.46 | 0.35 | 0.44 | 0.45 | 0 | 0.40 | 0.19 | 0.13 | 0.11 | 0.16 |
| 1 | 0.56 | 0.54 | 0.65 | 0.56 | 0.55 | 1 | 0.42 | 0.18 | 0.20 | 0.12 | 0.07 |
| The government pays for producers from tax funds | | | | |  | The government pays for producers from tax funds | | | | |  |
| 0 | 0.54 | 0.58 | 0.51 | 0.53 | 0.57 | 0 | 0.39 | 0.19 | 0.15 | 0.11 | 0.15 |
| 1 | 0.46 | 0.42 | 0.49 | 0.47 | 0.44 | 1 | 0.44 | 0.18 | 0.19 | 0.13 | 0.06 |
| Increased VAT in FAW products, which is allocated to producers in the system | | | | | | Increased VAT in FAW products, which is allocated to producers in the system | | | | | |
| 0 | 0.66 | 0.66 | 0.48 | 0.59 | 0.63 | 0 | 0.43 | 0.20 | 0.13 | 0.11 | 0.13 |
| 1 | 0.34 | 0.34 | 0.52 | 0.41 | 0.37 | 1 | 0.38 | 0.17 | 0.24 | 0.13 | 0.08 |
| *Information source considered trusted*: Good from Finland - organization | | | | | | *Information source considered trusted*: Good from Finland - organization | | | | | |
| 0 | 0.33 | 0.34 | 0.28 | 0.32 | 0.35 | 0 | 0.39 | 0.18 | 0.14 | 0.11 | 0.18 |
| 1 | 0.67 | 0.66 | 0.72 | 0.68 | 0.66 | 1 | 0.43 | 0.19 | 0.19 | 0.12 | 0.08 |
| Food processing companies | | |  |  |  | Food processing companies | | |  |  |  |
| 0 | 0.66 | 0.69 | 0.60 | 0.76 | 0.70 | 0 | 0.39 | 0.18 | 0.15 | 0.13 | 0.15 |
| 1 | 0.34 | 0.31 | 0.40 | 0.24 | 0.30 | 1 | 0.47 | 0.19 | 0.22 | 0.09 | 0.03 |
| Animal protection organization | | | |  |  | Animal protection organization | | | |  |  |
| 0 | 0.55 | 0.54 | 0.36 | 0.35 | 0.50 | 0 | 0.46 | 0.20 | 0.12 | 0.08 | 0.13 |
| 1 | 0.45 | 0.46 | 0.64 | 0.65 | 0.50 | 1 | 0.37 | 0.17 | 0.22 | 0.15 | 0.09 |
| Authorities | |  |  |  |  | Authorities | |  |  |  |  |
| 0 | 0.38 | 0.33 | 0.41 | 0.35 | 0.40 | 0 | 0.40 | 0.15 | 0.18 | 0.10 | 0.17 |
| 1 | 0.62 | 0.67 | 0.59 | 0.65 | 0.60 | 1 | 0.42 | 0.21 | 0.17 | 0.13 | 0.08 |
| Producers | |  |  |  |  | Producers | |  |  |  |  |
| 0 | 0.48 | 0.47 | 0.38 | 0.57 | 0.49 | 0 | 0.40 | 0.18 | 0.13 | 0.14 | 0.16 |
| 1 | 0.52 | 0.53 | 0.62 | 0.43 | 0.51 | 1 | 0.42 | 0.19 | 0.21 | 0.10 | 0.07 |
| Retail companies and organizations | | | |  |  | Retail companies and organizations | | | |  |  |
| 0 | 0.62 | 0.63 | 0.53 | 0.66 | 0.64 | 0 | 0.40 | 0.18 | 0.14 | 0.12 | 0.15 |
| 1 | 0.38 | 0.37 | 0.47 | 0.34 | 0.36 | 1 | 0.44 | 0.19 | 0.22 | 0.11 | 0.04 |
| Company established for FAW label system | | | | |  | Company established for FAW label system | | | | |  |
| 0 | 0.68 | 0.67 | 0.61 | 0.71 | 0.68 | 0 | 0.41 | 0.18 | 0.15 | 0.12 | 0.13 |
| 1 | 0.32 | 0.33 | 0.39 | 0.29 | 0.32 | 1 | 0.42 | 0.19 | 0.21 | 0.11 | 0.07 |
| *Knowledge of and connections with farms***:** No connections with animal farms | | | |  |  | *Knowledge of and connections with farms***:** No connections with animal farms | | | |  |  |
| 0 | 0.54 | 0.57 | 0.67 | 0.42 | 0.55 | 0 | 0.41 | 0.19 | 0.21 | 0.09 | 0.10 |
| 1 | 0.46 | 0.43 | 0.33 | 0.58 | 0.45 | 1 | 0.42 | 0.18 | 0.13 | 0.15 | 0.13 |
| Considers themselves to know (quite) a lot about animal production | | | | | | Considers themselves to know (quite) a lot about animal production | | | | | |
| 0 | 0.83 | 0.84 | 0.80 | 0.89 | 0.84 | 0 | 0.41 | 0.19 | 0.16 | 0.12 | 0.11 |
| 1 | 0.17 | 0.16 | 0.20 | 0.11 | 0.17 | 1 | 0.43 | 0.18 | 0.20 | 0.08 | 0.11 |
| Lived on production farms | | |  |  |  | Lived on production farms | | |  |  |  |
| 0 | 0.86 | 0.89 | 0.74 | 0.91 | 0.86 | 0 | 0.42 | 0.19 | 0.15 | 0.12 | 0.12 |
| 1 | 0.14 | 0.11 | 0.26 | 0.09 | 0.14 | 1 | 0.40 | 0.15 | 0.32 | 0.08 | 0.05 |

Appendix 3.

The BIC and CAIC values support the choice of 5 classes.

BIC, AIC and CAIC values for 1-8 classes choices. The values in bold indicate the lowest values.

| Number of classes |  | BIC(LL) | AIC(LL) | CAIC(LL) |
| --- | --- | --- | --- | --- |
| 1-Class Choice |  | 6131.245 | 6091.33 | 6141.245 |
| 2-Class Choice |  | 5514.858 | 5431.037 | 5535.858 |
| 3-Class Choice |  | 5256.977 | 5129.251 | 5288.977 |
| 4-Class Choice |  | 5222.624 | 5050.991 | **5265.624** |
| 5-Class Choice |  | **5211.654** | 4996.115 | **5265.654** |
| 6-Class Choice |  | 5217.272 | 4957.827 | 5282.272 |
| 7-Class Choice |  | 5228.709 | 4925.358 | 5304.709 |
| 8-Class Choice |  | 5255.928 | **4908.671** | 5342.928 |

Appendix 4. The probability (0,0-1,0) to belong to the class 1, 2, 3, 4 and 5.

Probability

Probability

Appendix 5A) Share of respondents choosing given price levels (€ cents) by groups 1, 2, 3, 4 and 5

cents € (/€)

5B) Share of respondents always choosing the product with higher AW (0-opt-outs), 1-2, 3-4 and 5-6 opt-out options (out of total six choice situations) by groups 1, 2, 3, 4 and 5.

| Number of opt-outs chosen out of six choice situations (by a respondent) | Group 1 | Group 2 | Group 3 | Group 4 | Group 5 |
| --- | --- | --- | --- | --- | --- |
| 0 opt-outs – product with higher FAW always chosen | 80 % | 45 % | 100 % | 0 % | 0 % |
| 1-2 opt-out options | 20 % | 41 % | 0 % | 55 % | 0 % |
| 3-4 opt-out options | 0 % | 13 % | 0 % | 45 % | 2 % |
| 5-6 opt-out options | 0 % | 0 % | 0 % | 0 % | 98 % |
